# Molecular characterization of *Cryptosporidium* and *Giardia* from the Tasmanian devil (*Sarcophilus harrisii*)

**DOI:** 10.1371/journal.pone.0174994

**Published:** 2017-04-19

**Authors:** Liana F. Wait, Samantha Fox, Sarah Peck, Michelle L. Power

**Affiliations:** 1 Department of Biological Sciences, Macquarie University, North Ryde, NSW, Australia; 2 Save the Tasmanian Devil Program, The Department of Primary Industries, Parks, Water and Environment, Hobart, Tasmania, Australia; Institut national de la santé et de la recherche médicale—Institut Cochin, FRANCE

## Abstract

The Tasmanian devil (*Sarcophilus harrisii*) is a carnivorous marsupial found only in the wild in Tasmania, Australia. Tasmanian devils are classified as endangered and are currently threatened by devil facial tumour disease, a lethal transmissible cancer that has decimated the wild population in Tasmania. To prevent extinction of Tasmanian devils, conservation management was implemented in 2003 under the Save the Tasmanian Devil Program. This study aimed to assess if conservation management was altering the interactions between Tasmanian devils and their parasites. Molecular tools were used to investigate the prevalence and diversity of two protozoan parasites, *Cryptosporidium* and *Giardia*, in Tasmanian devils. A comparison of parasite prevalence between wild and captive Tasmanian devils showed that both *Cryptosporidium* and *Giardia* were significantly more prevalent in wild devils (*p* < 0.05); *Cryptosporidium* was identified in 37.9% of wild devils but only 10.7% of captive devils, while *Giardia* was identified in 24.1% of wild devils but only 0.82% of captive devils. Molecular analysis identified the presence of novel genotypes of both *Cryptosporidium* and *Giardia*. The novel *Cryptosporidium* genotype was 98.1% similar at the 18S rDNA to *Cryptosporidium varanii* (syn. *C*. *saurophilum)* with additional samples identified as *C*. *fayeri*, *C*. *muris*, and *C*. *galli*. Two novel *Giardia* genotypes, TD genotype 1 and TD genotype 2, were similar to *G*. *duodenalis* from dogs (94.4%) and a *Giardia* assemblage A isolate from humans (86.9%). *Giardia duodenalis* BIV, a zoonotic genotype of *Giardia*, was also identified in a single captive Tasmanian devil. These findings suggest that conservation management may be altering host-parasite interactions in the Tasmanian devil, and the presence of *G*. *duodenalis* BIV in a captive devil points to possible human-devil parasite transmission.

## Introduction

*Cryptosporidium* and *Giardia* are two ubiquitous protozoan parasites capable of infecting a wide range of vertebrate species, including humans. There are multiple species and genotypes of *Cryptosporidium* and *Giardia*, and these have varying host-specificity and pathogenicity. Most species of *Cryptosporidium* and *Giardia* are morphologically indistinguishable, and hence molecular tools have been instrumental in allowing for species identification and differentiation. Both parasites are transmitted via direct contact with an infected host or through ingestion of contaminated food or water. *Cryptosporidium* and *Giardia* are of public health and agricultural significance as causes of enteric disease in humans and domestic animals. Studies of both parasites in wildlife have generally focused on ascertaining whether wildlife hosts might act as disease reservoirs for humans or domestic animals [1–3]. As such, little is known about the impact of *Cryptosporidium* and *Giardia* on wildlife species themselves. However, the ubiquity of *Cryptosporidium* and *Giardia*, along with the presence of species with both broad and narrow host-specificity within each genus, make them useful indicators for interactions between humans, domestic and wild animals.

*Cryptosporidium* has been identified in 16 marsupial species worldwide, including 14 Australian marsupials [4]. Two marsupial specific *Cryptosporidium* species, *C*. *fayeri* and *C*. *macropodum*, have been characterized from Australian marsupials, and multiple genotypes have also been found [5–9]. Estimates of the prevalence of *Cryptosporidium* in populations of Australian marsupials range from 6.7% to 12.2% [6, 8–11]. Differences in prevalence have been noted within populations during different seasons [10], and higher prevalence rates have been reported in populations from urbanised or agricultural settings [6, 11]. *Cryptosporidium* has been detected in both wild and captive marsupials [4], but only one study has directly compared wild and captive populations, finding no significant difference in *Cryptosporidium* prevalence between captive and wild brush-tailed rock wallaby (*Petrogale pencillata*) populations [9].

*Giardia* has been identified in many species of Australian marsupial, and molecular studies have allowed identification of the exact species present in marsupials. *G*. *duodenalis* has been the species identified in all except one of these molecular studies [11–13]; the exception being the finding of a host-specific species of *Giardia*, named *G*. *peramelis*, in the quenda (*Isoodon obsulus*) [14]. Estimates of the prevalence of *Giardia* in Australian marsupials range from 1.3% to 13.8% [8, 13, 15, 16]. No significant differences in the prevalence of *Giardia* have been found between captive and wild marsupial populations studied to date [12, 13].

This study investigated the prevalence and diversity of *Cryptosporidium* and *Giardia* in an endangered Australian marsupial, the Tasmanian devil (*Sarcophilus harrisii*). The Tasmanian devil is the largest extant carnivorous marsupial. Though once prevalent across mainland Australia, Tasmanian devils have been restricted to the island of Tasmanian for the last 3000–4000 years [17]. Devils can be found state-wide in Tasmania, though their habitat is becoming increasingly fragmented by urban and agricultural development. Tasmanian devils are currently threatened by a lethal transmissible tumour, devil facial tumour disease (DFTD)which was first discovered in 1996 and has since spread through more than 85% of the Tasmanian devil population [18, 19]. This rapid and severe decline resulted in the devil being listed as endangered in 2008 under the IUCN’s Red List and under the Tasmanian State Governments Threatened Species Protection Act, and in 2009 under the Australian Commonwealth Government’s Environmental Protection Biodiversity Conservation act. Due to the serious threat posed by DFTD, Tasmanian devils are subject to a conservation management program known as the Save the Tasmanian Devil Program (STDP). The primary goal of the program has been to establish a captive insurance population of genetically diverse and DFTD-free devils. Tasmanian devils in the insurance population are housed in a range of intensive enclosures and free-range facilities in Tasmania and on mainland Australia. The recovery program has also begun to establish wild populations of devils on DFTD-free islands and peninsulas off of Tasmania. These wild populations are expected to provide multiple benefits, including allowing devils to maintain wild behaviours and to interact with the natural ecosystem. Establishment of DTFD-free wild populations also facilitates one of the conservation program’s other goals: to conserve commensal, symbiotic and parasitic organisms associated with Tasmanian devils [20].

Tasmanian devils are known to host a variety of parasites, including nematodes, cestodes, digenea, ectoparasites and protozoa [21]. Two protozoal genera, *Giardia* and *Sarcocystis*, have been detected in Tasmanian devils, but studies of these parasites in this host have relied on classical techniques, and have therefore not been able to identify the parasites beyond a genus level [22–24]. Two such studies have reported on the prevalence of *Giardia* in Tasmanian devils with the first estimate of 8.3% (*n* = 12) [24] and the second 6.0% (*n* = 32) [23]. An unpublished report identified a novel genotype of *Giardia* from a Tasmanian devil [1], however there is not genetic data for this genotype on GenBank, nor is there information on the origin of the host. Antibodies specific to a third protozoan, *Toxoplasma gondii*, have been detected in the blood of Tasmanian devils, but parasite stages themselves have not been isolated from devils [25]. *Cryptosporidium* has never been reported from Tasmanian devils.

The aims of this study were (1) to characterize *Cryptosporidium* and *Giardia* species in Tasmanian devils, and (2) to determine whether conservation management may be changing the prevalence and diversity of *Cryptosporidium* and *Giardia* in Tasmanian devils. To achieve this, molecular typing was used to characterize *Cryptosporidium* and *Giardia* from three different Tasmanian devil population types with varying levels of human contact: intensively managed captive populations, free-range captive populations, and wild populations of devils. Additionally, pre- and post-release faecal samples were analysed from a group of captive devils that were released into the wild in order to examine potential changes to the parasite community as a result of re-wilding.

## Materials and methods

### Sample collection

A total of 216 faecal samples were collected from 190 Tasmanian devils (Table 1). The sample populations consisted of five intensively managed captive populations (Monarto Zoo, Healesville Sanctuary, Taronga Zoo, Western Plains Zoo, and the Cressy STDP breeding facility), three free-range captive populations (Devil Ark and the STDP free-range enclosures at Bridport and on the Freycinet Peninsula), and three wild populations (Table Mountain, Narawntapu National Park and Kempton). All samples were collected between July 2015 and May 2016, with the exception of 18 samples from Devil Ark that were collected in June 2014. Wild samples were collected from July 2015–May 2016, free-range captive samples were collected from November 2015–February 2016, and intensive captive samples were collected from October 2015–February 2016. Additionally, pre- and post-release faecal samples were collected from 16 devils that were released into Narawntapu National Park in September 2015. All released devils were either bred in captivity as part of the insurance population or had been housed in captivity for at least three years prior to the trial, and were housed in the Freycinet and TasZoo free-range captive enclosures for 4–6 months prior to release. Pre-release samples were taken directly prior to release of devils and post-release samples were collected during trapping trips in Narawntapu National Park approximately 2, 4, 8, and 12 weeks following release.

**Table 1:** Results and species identification for *Cryptosporidium* and *Giardia* in different populations of Tasmanian devils. For the released devils, the 13 pre-release samples each represent a different individual, while the nine post-release samples came from four individuals, three of which did not provide a pre-release sample.

			*Cryptosporidium*				*Giardia*			
Population Type	Population		No. of faecal samples	No. of animals	18S rDNA	*actin*	No. of faecal samples	No. of animals	*B-giardin*	*gdh*
**Intensive Captive**	Monarto Zoo		7	7	0	0	7	7	0	0
	Healesville Sanctuary		20	20	3	0	20	20	0	0
	Taronga Zoo		3	3	0	0	3	3	0	0
	Western Plains Zoo		10	10	0	0	10	10	0	0
	Cressy facility		18	18	2	0	18	18	0	0
		Total	58	58	5	0	58	58	0	0
**Free range captive**	Devil Ark		36	36	2	0	36	36	0	0
	Freycinet Peninsula		14	12	0	0	14	12	0	1
	Bridport		22	16	6(8)*	0	22	16	0	0
		Total	72	64	10	0	72	64	0	1
**Wild**	Narawntapu National Park		38	25	10	0	32	21	1	5
	Table Mountain		8	8	4	1	8	8	0	2
	Kempton		18	18	9	0	-	-	-	-
		Total	64	51	23	1	40	29	1	7
**Released devils**	Pre-release		13	13	1	0	13	13	0	0
	Post-release		9	4 (3 new)	2	1	8	4 (3 new)	0	0
		Total	22	17	3	1	21	17	0	0
		**Total**	216	190	41	191	167	167	1	8

All samples collected from wild field sites were obtained with permission from the relevant authority. Permission to work in Narawntapu National Park was granted by the Tasmanian Parks and Wildlife Service. Table Mountain is private land, and permission was given by the land holder. Kempton is owned by a Forestry Company (Forico), who granted permission to work on their land. All activities carried out in the field were covered by a permit issued by the Department of Primary Industries, Parks, Water and the Environment, a department of the Tasmanian State Government.

Faecal samples were collected opportunistically and non-invasively. Scat collection from the captive facilities was carried out during routine husbandry maintenance days. For the wild sites, scats were collected opportunistically when devils defecated in traps or during handling. This routine trapping was covered by a Standard Operating Procedure, lodged with the Departmental Ethics Advisory group (08/2014-15). Where possible, the identity, sex and age of the animal were noted for each sample. For samples collected during daily cleaning from group pens, only a single sample was collected in order to prevent re-sampling. Following collection, all samples were stored at 4°C.

### DNA extraction and general PCR methods

DNA was extracted from ~150 mg of each faecal sample using the Isolate Fecal DNA kit (Bioline, London, UK) according to the manufacturer’s instructions. Extracted DNA was stored at -20°C. All PCR’s were performed in conjunction with a negative control (sterile H_2_O) and a positive control (DNA extracted from purified *C*. *parvum* oocysts or *G*. *lamblia* cysts acquired from Waterborne Inc, USA). For all protocols, secondary PCR products were resolved by electrophoresis on a 1.5% agarose gel containing 2μL of SYBR Safe DNA Gel Stain (Invitrogen, Carlsbad, California) and visualized under UV light. Bands were compared to a Hyperladder II DNA marker (Bioline, London, UK) to estimate amplicon size. PCRs were performed in an Eppendorf Mastercycler (Eppendorf, North Ryde, Australia).

### Cryptosporidium PCR: 18S rDNA, actin and gp60 loci

For all *Cryptosporidium* protocols, samples were combined with an equal volume of GeneReleaser (BioVentures, Inc., TN, USA) and microwaved for seven minutes in a 500W microwave directly prior to PCR analysis. Samples were initially screened for *Cryptosporidium* at the 18S rDNA locus (~825 bp) using a previously described nested PCR [9]. Primary and secondary reaction mixtures contained 2 mM MgCl_2_, 200 μM dNTPs, 200 nM of each primer, and 2.5 U of Red Hot Taq DNA polymerase (Thermo Scientific, Australia). Reaction conditions comprised an initial denaturation of 94°C for 3 min, followed by 35 cycles of 94°C for 45 s, 56°C for 45 s, and 72°C for 1 min, with a final extension at 72°C for 7 min.

In order to confirm positive status and for phylogenetic analysis, samples found positive for *Cryptosporidium* at the 18S rDNA locus were screened at the *actin* locus (~800 bp) using the primers from a previously described nested PCR [26] with previously introduced modifications [9]. Primary and secondary reaction mixtures contained 2 mM MgCl_2_, 200 μM dNTPs, 2.5 U of Red Hot Taq DNA polymerase (Thermo Scientific, Australia), and 200 nM of each primer. Reaction conditions comprised an initial denaturation of 94°C for 5 min, followed by 35 cycles of 94°C for 45 s, 50°C for 45 s, and 72°C for 1 min, with a final extension at 72°C for 10 min. The conditions for the secondary PCR were identical, except for a higher annealing temperature (54°C).

Positive samples were also screened at the *gp60* locus (~1000 bp) using a previously described nested PCR [9, 26]. Primary and secondary reaction mixtures contained 4 mM MgCl_2_, 200 nM dNTPs, 200 nM of each primer, and 1 U of Red Hot Taq DNA polymerase (Thermo Scientific, Australia). Reaction conditions comprised an initial denaturation at 94°C for 3 min followed by 35 cycles of 94°C 45 s, 58°C 45 s and 72°C for 1 min 30 s, with a final extension at 72°C for 5 min.

### Giardia PCR: β-giardin and gdh loci

DNA from 191 faecal samples was screened for *Giardia* at the *β-giardin* (~511 bp) and *gdh* (~432 bp) loci. The *β-giardin* locus was tested using a previously described nested PCR [27] with a modified secondary forward primer [28]. Primary and secondary reaction mixtures contained 1.5 mM MgCl_2_, 200 nM dNTPs, 10 pmol of each primer, and 1 U of Tth DNA polymerase (Promega, USA). Reactions conditions comprised an initial denaturation at 95°C for 15 min followed by 35 cycles of 95°C for 30 s, 65°C for 30 s and 72°C for 1 min 30 s, with a final extension at 72°C for 7 min.

Testing at the *gdh* locus used a previously described semi-nested PCR [29]. Primary and secondary reaction mixtures contained 1.5 mM MgCl_2_, 200 nM dNTPs, 12.5 pmol of each primer, and 1 U of Tth DNA polymerase (Promega, USA). Reactions conditions comprised an initial cycle of 94°C for 2 min, 56°C for 1 min and 72°C for 2 min, 55 cycles of 94°C for 30 s, 56°C for 20 s and 72°C for 45 s, with a final extension at 72°C for 7 min.

### DNA sequencing, sequence analysis, and phylogenetic analysis

Amplicons for all *Cryptosporidium* and *Giardia* loci were purified using the QIAquick PCR Purification Kit (Qiagen, Hilden, Germany) and sequenced in the forward and reverse directions (Ramaciotti Centre for Genomics, Randwick, Australia). Forward and reverse sequences were aligned and assessed manually for quality, and consensus sequences were extracted using Geneious version 8.0.5 (Biomatters, New Zealand). BLAST searches were conducted on consensus sequences for genus confirmation and species identification.

Based on Blast identifications, a consensus sequence of a novel *Cryptosporidium* genotype was generated. The novel sequences and additional sequences identified as *Cryptosporidium* species were aligned to *Cryptosporidium* reference sequences from GenBank using ClustalW [30]. The process for *Cryptosporidium* sequences was also applied to *Giardia* sequences. The alignments for both parasites were trimmed to equal sequence length. A phylogeny was inferred using Bayesian Markov Chain Monte Carlo (MCMC) analysis using a general time reversible (GTR) model and a gamma distribution with four rate categories. The Bayesian tree was created in Geneious version 8.1.3 using Mr. Bayes 3.2.2 [31] with 4 MCMC heated chains, a chain length of 5 million with a subsampling frequency of 1,000 and a burn-in length of 100,000 [32].

DNA sequences for *Cryptosporidium* genotypes were lodged in GenBank under accession numbers KY490553—KY490556 (18S rDNA sequences) and KY618125 – KY618126 (*actin* sequence) and for *Giardia* under KY618122 – KY618124 (*gdh* sequences) and KY652163 (*β-giardin*).

### Statistics

Statistical analyses were conducted using R version 3.2.5 [33]. Prevalence estimates were calculated by dividing the number of positive samples by the number of devils in the relevant population. For calculating prevalence, only the first sampling event was included for devils with repeat samples. The prevalence of *Cryptosporidium* and *Giardia* was compared between population types and between facilities within each population type using a two-tailed Fisher exact test.

## Results

### Cryptosporidium

#### Prevalence of *Cryptosporidium* in the Tasmanian devil

PCR screening identified 41/216 samples as positive for *Cryptosporidium* at the 18S rDNA locus (Table 1). These 41 samples came from 39 individual animals: two juvenile Tasmanian devils from Bridport free-range enclosure tested positive twice, with 21 days in-between sampling events. There was no significant difference in prevalence (*p* = 1.000) between the intensive captive (8.6%; 95% C.I. = 3.2–19.7%) and free-range captive population types (12.5%; 95% C.I. = 5.9–23.7%). However, the prevalence in the wild population (45.1%; 95% C.I. = 31.4–59.5%) was significantly higher than the prevalence in the captive population types (*p* < 0.001).

At the population level, there was no significant difference in prevalence (*p* = 0.840) among the Table Mountain, Narawntapu and Kempton wild populations and the Bridport free-range captive population (33.3–50.0%). Neither was there a significant difference (*p* = 0.676) in *Cryptosporidium* prevalence among the Freycinet free-range captive population, Devil Ark, Healesville, Cressy, Monarto, Taronga, and Western Plains Zoos populations (0.0–15.0%). However, the 37.5% prevalence of *Cryptosporidium* in the Bridport free-range population was significantly higher than the prevalence in the other captive populations (*p* = 0.002).

Information on sex was available for 174/216 samples that were tested for *Cryptosporidium*, of which 87 were from male Tasmanian devils and 87 from females. There was no significant difference (*p* = 0.222) in the prevalence of *Cryptosporidium* between samples from males (20.7%; C.I. = 13.0–31.0%) compared with females (12.6%; C.I. = 6.9–21.9%).

To confirm positive status and to allow for *Cryptosporidium* species identification, samples that were positive at the 18S rDNA locus were also screened at the *actin* and *gp60* loci. At the *actin* locus, 2/41 positive samples were confirmed positive for *Cryptosporidium*. None of the 41 samples that were positive at the 18S rDNA locus amplified at the *gp60* locus, despite a strong amplicon being produced for the *C*. *parvum* positive control.

#### *Cryptosporidium* species identification and phylogenetic analysis

Of the samples that were positive for *Cryptosporidium* at the 18S rDNA locus, 37/41 were 100% identical to each other and BLAST searches revealed that these samples represent a novel genotype of *Cryptosporidium*, from here on named *Cryptosporidium sp Tasmanian devil genotype*. *Cryptosporidium varanii* (syn. *C*. *saurophilum)* was the most similar species to *Cryptosporidium sp Tasmanian devil* genotype (98.1%). One further sample, for which only a partial reverse sequence was obtained, was identical to the novel genotype over a span of 429 nucleotides. Faecal samples containing *Cryptosporidium sp Tasmanian devil genotype* were from wild, intensive captive, and free-range captive population types, as well as from two released devils, 39 and 47 days post-release, respectively; no other samples were collected from the first of these devils, but for the second devil, a sample obtained 14 days post-release was negative for *Cryptosporidium*, as was a sample obtained 77 days post-release. The novel *Cryptosporidium* Tasmanian devil genotype was amplified on both occasions for the two juvenile devils that were positive for *Cryptosporidium* on re-sampling, with 21 days in between sampling events.

BLAST searches revealed that one of the 18S rDNA-positive samples (TD20, a wild devil from Table Mountain) was a 100% match to *C*. *fayeri* (GenBank: KP730318.1). The *actin* sequence for TD20 was also highly similar to *C*. *fayeri*, sharing 99.6% nucleotide identity with GenBank sequence KP730322.1. One other sample (TD63, a released devil, 47 days post-release, and identified as the novel *Cryptosporidium* Tasmanian devil genotype at the 18S rDNA locus) amplified at the *actin* locus, where it shared 99.5% nucleotide identity with *C*. *fayeri* (GenBank: KP730318.1).

Two further samples were identified as *C*. *muris* and *C*. *galli*. A wild Tasmanian devil sample (TD19) from Table Mountain was a 99.4% match for *C*. *muris* isolates (GenBank: KP994665.1, EU245044) and a sample from a pre-released devil (TD43) was a 99.8% match for *C*. *galli* (GenBank: KU744848.1).

**Fig 1:**
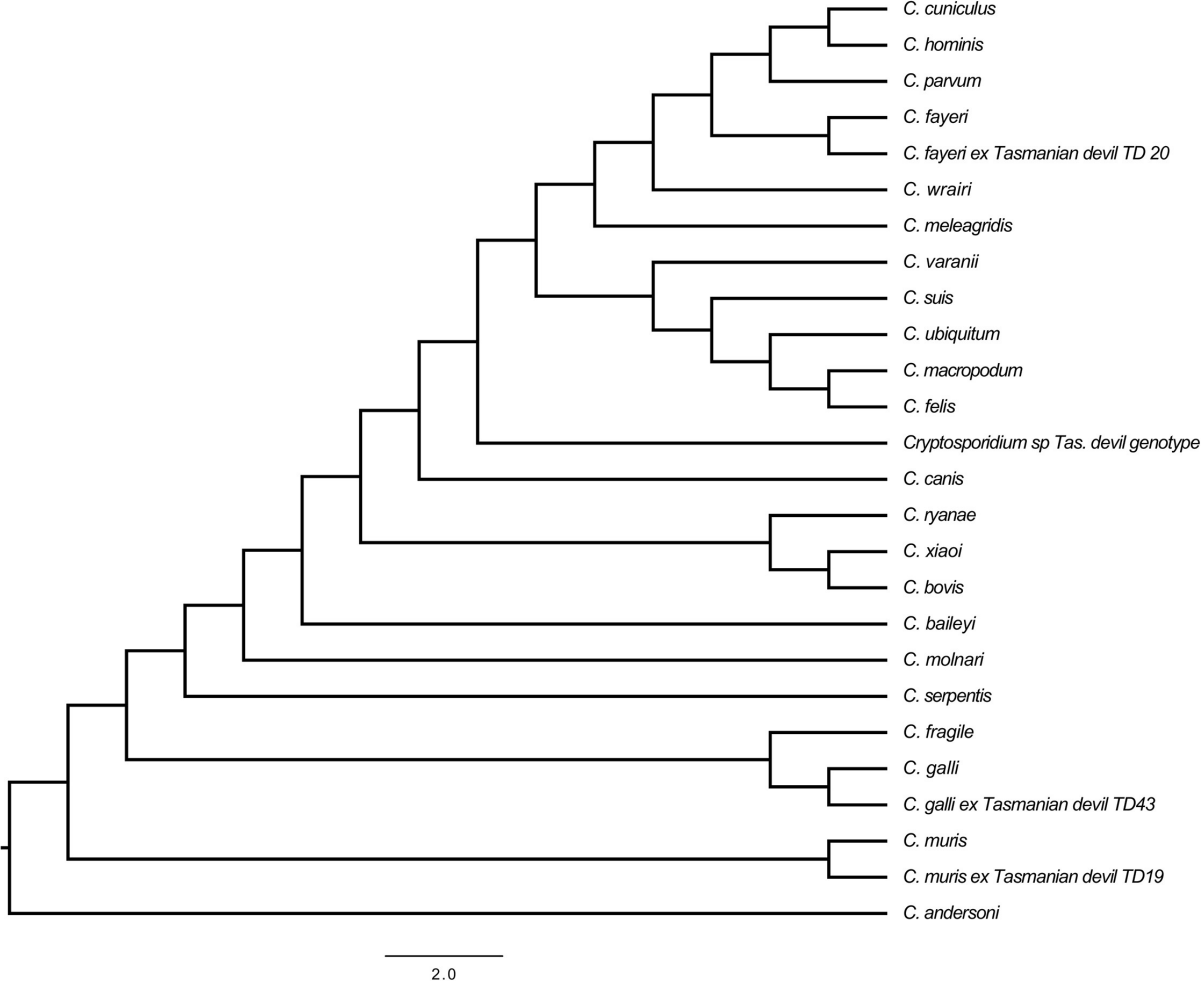
Inferred phylogeny of *Cryptosporidium* 18S rDNA consensus sequences (709 nucleotides). The *Cryptosporidium* 18S rDNA reference sequences used for this phylogeny are as follows: *C*. *andersoni* (FJ463171), *C*. *baileyi* (L19068), *C*. *bovis* (AY741305), *C*. *canis* (AF112576), *C*. *cuniculus* (FJ262765), *C*. *fayeri* (AF108860), *C*. *felis* (AF108862), *C*. *fragile* (EU162751), *C*. *galli* (HM116388), *C*. *hominis* (AF093491), *C*. *macropodum* (AF513227), *C*. *meleagridis* (AF112574), *C*. *molnari* (HM243548), *C*. *muris* (AF093497), *C*. *parvum* (AF108864), *C*. *ryanae* (FJ463193), *C*. *serpentis* (AF151376), *C*. *suis* (AF108861), *C*. *ubiquitum* (AF442484), *C*. *varanii* (AF112573), *C*. *wrairi* (AF115378), *C*. *xiaoi* (FJ896053).

Phylogenetic analyses placed each of the isolates in clades that aligned with their Blast identifications (Fig 1). The novel *Cryptosporidium* genotype from the Tasmanian devils was placed in the large clade containing intestinal species (Fig 1).

### Giardia

#### Prevalence of *Giardia* in Tasmanian devils

PCR screening identified 8/191 samples as positive for *Giardia* at either the *β-giardin* or *gdh* loci (Table 1). Of these eight samples, one amplified at both the *β-giardin* or *gdh* loci; the remaining seven positive samples amplified only at the *gdh* locus. The eight positive samples came from eight different devils. There was no significant difference (*p* = 1.000) in the prevalence of *Giardia* between the intensive captive (0.0%; 95% C.I. = 0.0–7.7%) and free-range captive population types (1.6%; 95% C.I. = 0.0–9.5%). However, the prevalence in the wild population type (24.1%; 95% C.I. = 11.0–43.9%) was significantly higher than the captive population types (*p* < 0.001). At the population level, there was no significant difference in *Giardia* prevalence (*p* = 1.000) between the Table Mountain and Narawntapu wild populations (8.3–25.0%). Likewise, there was no significant difference in prevalence (*p* = 0.262) between any of the captive populations (0.0–8.3%).

Information on sex was available for 149/191 samples tested for *Giardia*, of which 75 were from males and 72 from females. There was no significant difference (*p* = 1) between the prevalence of *Giardia* between males (2.7%; C.I. = 0.46–10.2%) and females (2.8%; C.I. = 0.48–10.6%).

#### *Giardia* species identification and phylogenetic analysis

**Fig 2:**
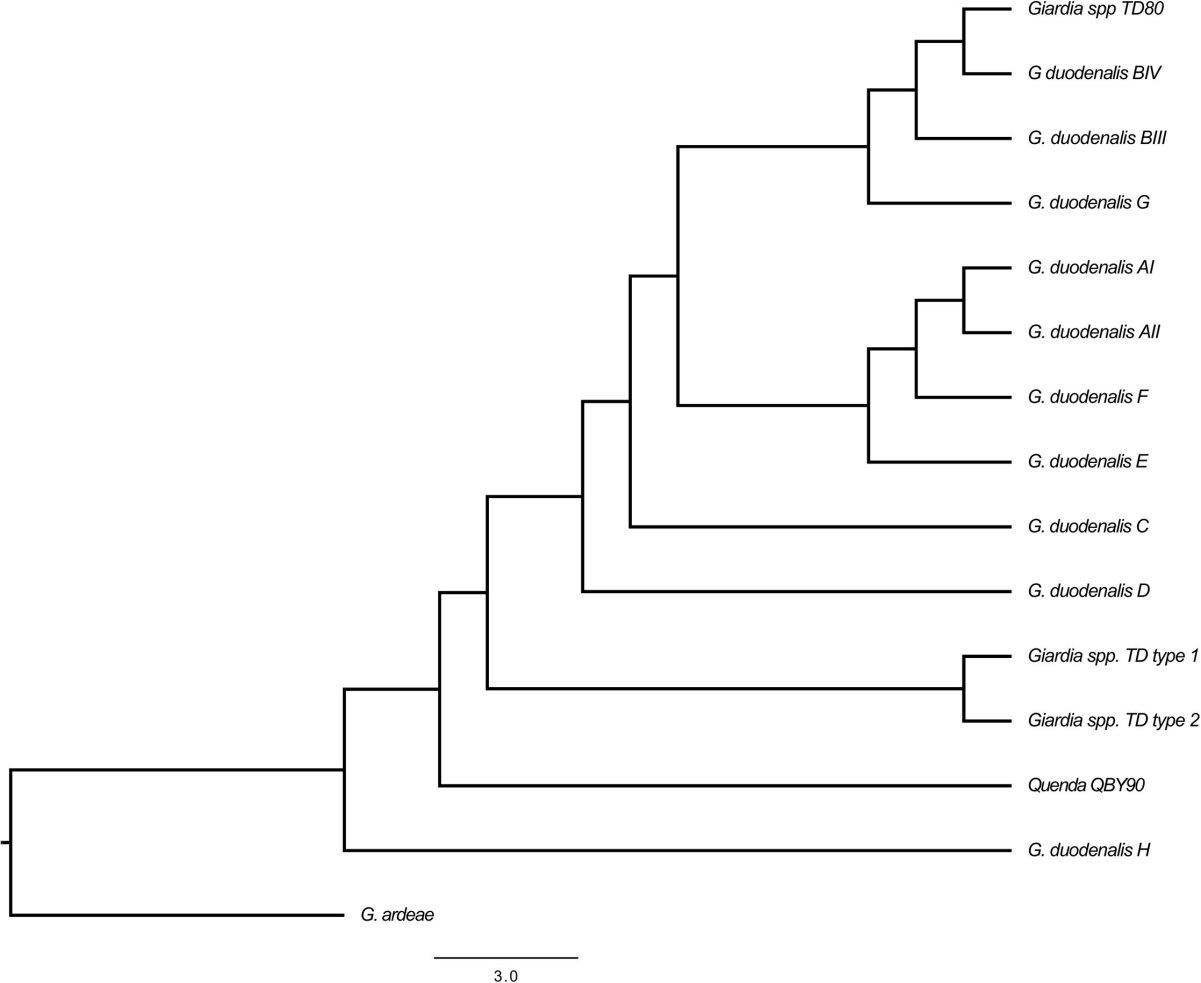
Inferred phylogeny of *Giardia gdh* sequences (199 nucleotides). The *Giardia gdh* reference sequences used for this phylogeny are as follows: *G*. *duodenalis* A1 (JN982015), *G*. *duodenalis* AII (L40510), *G*. *duodenalis* BIII (AF069059), *G*. *duodenalis* BIV (L40508), *G*. *duodenalis* C (U60983), *G*. *duodenalis* D (U60986), *G*. *duodenalis* E (DQ182601), *G*. *duodenalis* F AF069057, *G*. *duodenalis* H (GU176101.1), *G*. *ardeae* (AF069060). The Quenda_QBY90 sequence was obtained via personal communication with Alison Hillman and Amanda Ash at Murdoch University.

PCR screening at the *gdh* and *β-giardin* identified eight samples as positive for *Giardia* (Table 1). Four of the eight *gdh* positive samples were >99.6% identical to each other. BLASTN searches indicated that the four *gdh* positive samples represent a novel genotype of *Giardia (Giardia sp*. *TD type 1)*; overall these sequences from the Tasmanian devil were more similar to isolates from dogs (94.4%) (Genbank: U60982). The three remaining samples were identical to each other, with 100% nucleotide agreement. BLASTN analysis indicated that these three samples represent another novel genotype (*Giardia sp*. *TD type 2)*, with the closest match (86.9%) being *G*. *duodenalis*, assemblage A isolated from humans in South India (GenBank: JN616252.1). The nucleotide identity between the two novel *Giardia* sequences from Tasmanian devils was 94.3% over a stretch of 402 nucleotides. Phylogenetic analyses at the *gdh* locus grouped the two novel genotypes together in the main *G*. *duodenalis* clade and separately to other *Giardia* assemblages (Fig 2)

The final *gdh* positive sample (TD80) was found to be a 100.0% match to *G*. *duodenalis*, assemblage B (GenBank: JX448643.1). Alignment with GenBank reference sequences for *G*. *duodenalis* assemblage BIV revealed that this amplicon was only one nucleotide different from *G*. *duodenalis* BIV (GenBank: L40508). This sample originated from a devil in the Freycinet free-range captive population.

Only one sample, identified as *Giardia sp*. *TD type 1* at the *gdh* locus, was positive at the *β-giardin* locus. A BLAST search of sequence data found this sample to be a 91.4% match for *G*. *duodenalis* (GenBank: KP687756.1).

## Discussion

This study provides the first report of *Cryptosporidium*, and the first published molecular characterisation of *Giardia*, in Tasmanian devils. *Cryptosporidium* was detected in wild, intensive captive and free-range captive devils, while *Giardia* was only detected in wild and free-range captive devils. A novel genotype of *Cryptosporidium* was identified, along with three described species, *C*. *fayeri*, *C*. *muris*, and *C*. *galli*. Two novel genotypes of *Giardia* were identified, and *G*. *duodenalis* assemblage BIV was identified in a single free-range captive devil.

Both *Cryptosporidium* and *Giardia* were significantly more prevalent in wild compared with captive populations of Tasmanian devils. This could be due to several management factors. *Cryptosporidium* and *Giardia* have direct life cycles that rely on contact with infected faecal material or contaminated food or water for transmission (1). Captive devils in intensively managed facilities are sometimes housed individually which would preclude transmission via contact with faeces from infected individuals. Additionally, captive enclosures are routinely cleaned of faecal material in both intensive and free-range captive facilities, which would reduce the amount of infectious material available for parasite transmission. The lower occurrence of *Cryptosporidium* and *Giardia* in captive devils could have health implications for individuals that are subsequently released into the wild, as they may have little or no acquired immunity against parasites which they are likely to be exposed to in the wild. Further study of released Tasmanian devils and the changes that occur in their parasite communities following release are warranted to investigate this issue.

### Cryptosporidium

*Cryptosporidium* has previously been described in a range of Australian marsupials. This study found a 45.1% prevalence of *Cryptosporidium* in wild Tasmanian devils. This value is higher than prevalence estimates of *Cryptosporidium* in other Australian marsupials, which range from 6.7% to 12.2% [6, 8–11]. Screening at secondary loci resulted in low detection, with only two samples successfully amplified at the *actin* locus, and no samples amplified at the *gp60* locus. This failure to amplify at confirmatory loci may be due to both *actin* and *gp60* being single copy loci compared with the multi-copy 18S rDNA locus, resulting in low template DNA yield and poor amplification [6, 34]. Inefficient primer binding, as a result of mis-match to the novel *Cryptosporidium* genotype, may also account for inability to amplify at *actin* and *gp60* loci. Additionally, the *gp60* locus is highly polymorphic and only a few *Cryptosporidium* species to date have been characterized at this locus [34–37]. Future studies of *Cryptosporidium* in Tasmanian devils should test different primers sets for conventional loci, target different loci, and attempt to increase the amount of template DNA by isolating and concentrating oocysts after PCR detection in faecal DNA extracts.

The majority of *Cryptosporidium*-positive samples represent a novel genotype, named the *Cryptosporidium* sp. Tasmanian devil genotype. The finding of a novel genotype of *Cryptosporidium* in Tasmanian devils is not entirely unexpected; it is not unusual for novel genotypes of *Cryptosporidium* to be identified when wildlife hosts are investigated for *Cryptosporidium* for the first time [38, 39]. To further characterize this novel genotype, amplification and sequencing of other loci, and data on oocyst morphology are required.

The marsupial specific species *C*. *fayeri* was identified in one sample at the 18S rDNA locus, and two further samples were highly similar to *C*. *fayeri* at the *actin* locus. The latter sample was not identified as *C*. *fayeri* at the 18S rDNA locus, indicating a mixed infection in this individual. *C*. *fayeri* is a marsupial-specific species of *Cryptosporidium* that has been identified in a range of marsupial hosts from mainland Australia, including macropods, koalas, and bandicoots. This *Cryptosporidium* species is thought to be host-adapted, as the species is primarily found in marsupial hosts and infections in marsupials have not been associated with disease [4]. However, *C*. *fayeri* was identified as the cause of a human clinical case of cryptosporidiosis in 2009 [40], and the finding of this species in Tasmanian devils could have health implications for people working closely with devils.

*C*. *muris*, which was identified in a single Tasmanian devil sample, is a species of *Cryptosporidium* with broad host-specificity. Rodents (*Mus musculus* and *Rattus spp*.) serve as the primary hosts for *C*. *muris* [41], and this species also infects immunocompromised humans and a range of other eutherian mammals [1]. Notably, *C*. *muris* has been documented in one other Australian marsupial, the bilby (*Macrotis lagotis*) [42]; in this case, multiple infections occurred in a captive colony of bilbies and were traced to house mice entering the colony [42]. *C*. *muris* is not a genetically uniform species, and studies have identified multiple genetically distinct subtypes [26, 43, 44]. The 0.7% variation found between the isolate identified here and recorded isolates of *C*. *muris* is comparable to the amount of variation seen between other recorded isolates of *C*. *muris* at the 18S rDNA locus.

*Cryptosporidium galli*, a species that causes clinical disease in birds [45], was identified in one Tasmanian devil sample. This is the first report of *C*. *galli* in a mammalian host. The sample did not amplify at *actin* or *gp60* and additional analyses should be undertaken to confirm this finding. Though this would be the first example of *C*. *galli* in a mammalian host, *C*. *meleagridis*, a species of *Cryptosporidium* that infects birds and humans [1], has recently been identified in the brush-tailed rock wallaby (*Petrogale pencillata*), another Australian marsupial [9]. It is possible that *C*. *galli* DNA may have been amplified from a devil passively passaging oocysts from feed sources and not from infection. Captive devils are occasionally fed chicken (pers. comm. Olivia Barnard), and are also exposed to wild birds in their enclosures, two possible transmission or contamination routes for *C*. *galli*.

### Giardia

*Giardia* has previously been described in a range of Australian marsupials. Our estimate of prevalence of *Giardia* in wild Tasmanian devils (24.1%) is higher than prevalence estimates for mainland Australian marsupials (range 1.3–13.8%) [8, 13, 15, 16], but sits within the range of prevalence estimates for Tasmanian marsupials (6.25–61.5%) [46]. Our 24.1% prevalence estimate for wild devils was also higher than previous prevalence estimates of *Giardia* in devils of 8.3% (*n* = 12) [24] and 6.0% (*n* = 32) [23]. This higher estimate is likely due to the use of molecular techniques in this study, which are more sensitive than faecal flotation methods [47, 48] used in prior studies of *Giardia* in Tasmanian devils [23, 24].

Molecular studies of *Giardia* in other Australian marsupials have found that the quenda (*Isoodon obesulus*) hosts a novel species, *G*. *peramelis*, while other Australian marsupials have been shown to host various assemblages of *G*. *duodenalis* (syn. *G*. *lamblia* and *G*. *intestinalis*) [11–14]. *G*. *duodenalis* is the causative agent of human giardiasis, and rather than representing a single species, molecular studies of *G*. *duodenalis* have revealed a species-complex that is divided into eight genetic assemblages (A-H). The assemblages are further divided into sub-assemblages (ie. AI, AII) with varying pathogenicity and host-specificity [3]. This study identified two novel genotypes of *Giardia* in Tasmanian devils, and identified the zoonotic assemblage *G*. *duodenalis* BIV in a single captive devil. Further work is required to characterise the two novel genotypes. Cyst morphometrics, in combination with molecular analysis at other loci such as the ITS1-5.8s-ITS2 locus, would enable comparison to *G*. *peramelis*, the *Giardia* species found in the quenda [14, 16]. The zoonotic assemblage *G*. *duodenalis* BIV has previously been described in a number of Australian marsupial species, including another species of dasyurid, the spotted-tailed quoll (*D*. *maculatus*) [12, 13]. The identification of this assemblage in a Tasmanian devil could imply spill-over from humans to Tasmanian devils. Such human-devil transmission could result from direct contact, but would more likely be the result of human environmental contamination.

## Conclusions

This study reports the presence of novel genotypes of *Cryptosporidium* and *Giardia* in Tasmanian devils. A comparison of parasite prevalence between captive and wild devils showed a significantly higher prevalence of both *Cryptosporidium* and *Giardia* in wild devils. Isolates with high similarity to *C*. *fayeri*, *C*. *muris*, and *C*. *galli* were also identified. *G*. *duodenalis* BIV, a zoonotic genotype of *Giardia*, was identified in a captive devil, indicating that human-devil transmission may be occurring. Further investigation of *Giardia* in captive Tasmanian devils is warranted, as is investigation of other possible human-specific pathogens. Overall, the findings suggest that captive management may be changing host-parasite interactions in the Tasmanian devil, as evidenced by the lower prevalence of both *Cryptosporidium* and *Giardia* in captive compared with wild devils. These findings should help to guide the Save the Tasmanian Devil Program in their goal to conserve devil-associated symbionts and parasites.
